# BLOND, a building-level office environment dataset of typical electrical appliances

**DOI:** 10.1038/sdata.2018.48

**Published:** 2018-03-27

**Authors:** Thomas Kriechbaumer, Hans-Arno Jacobsen

**Affiliations:** 1 Department of Computer Science, Chair for Application and Middleware Systems, Technical University of Munich, 85748 Garching, Germany

**Keywords:** Energy and behaviour, Energy modelling, Electrical and electronic engineering

## Abstract

Energy metering has gained popularity as conventional meters are replaced by electronic smart meters that promise energy savings and higher comfort levels for occupants. Achieving these goals requires a deeper understanding of consumption patterns to reduce the energy footprint: load profile forecasting, power disaggregation, appliance identification, startup event detection, etc. Publicly available datasets are used to test, verify, and benchmark possible solutions to these problems. For this purpose, we present the BLOND dataset: continuous energy measurements of a typical office environment at high sampling rates with common appliances and load profiles. We provide voltage and current readings for aggregated circuits and matching fully-labeled ground truth data (individual appliance measurements). The dataset contains 53 appliances (16 classes) in a 3-phase power grid. BLOND-50 contains 213 days of measurements sampled at 50kSps (aggregate) and 6.4kSps (individual appliances). BLOND-250 consists of the same setup: 50 days, 250kSps (aggregate), 50kSps (individual appliances). These are the longest continuous measurements at such high sampling rates and fully-labeled ground truth we are aware of.

## Background & Summary

Electrical energy metering (EEM) has experienced an influx of research activity in recent years due to the shift from mechanical to electronic metering technology. Metering devices used for measuring electrical energy consumption (EEC) and billing consumers are subjected to increased scrutiny over accuracy and reliability. The migration to fully digital EEM is often motivated by energy saving promises and higher comfort levels for occupants. EEC profiles can be generated in smaller time intervals (e.g., daily, hourly, by minute), since smart meters allow for automated meter readings.

Recent studies into the psychological effects of EEM feedback have shown that saving energy and actively managing one's EEC requires frequent feedback over long periods, ideally with an appliance-specific breakdown^1^. However, this requires a significant investment in metering hardware, infrastructure, and reliable communication channels to collect the data from a fleet of smaller meters. Non-intrusive appliance load monitoring (NIALM) attempts to solve this by relying on single-point EEM, ideally utilizing an existing smart meter, to provide a disaggregated view of the whole-building EEC^2^. Researchers make use of public datasets to study the characteristics of appliances and to build models representing load profiles and per-appliance usage. This can be beneficial for energy reduction^3,4^, pattern recognition^5–8^, energy demand forecasting^9^, and similar fields of study.

Existing datasets predominately cover household and residential environments^10–22^ due to the cost savings potential for their occupants. Large appliances (e.g., space heating, HVAC, washing machines, etc.) are being targeted first to achieve an immediate reduction in EEC since households typically contain a manageable number of them. These devices are easier to detect than multiple smaller ones, therefore, most datasets use measurement intervals of 1 sample per second (Sps), 1 min, or lower. Using sampling rates above 10kSps is beneficial to the total number and types of distinguishable appliances in a circuit with NIALM and appliance identification research questions^23^. The amount of information contained in electricity signals increases steadily with sampling rates ranging up to 1MHz. Higher sampling rates can capture subtle changes (high frequency ripples), which are useful for appliance identification^5,23–25^. Capturing the voltage and current waveforms allows energy disaggregation algorithms such as BOLT^6^ to extract patterns directly from the raw measurement data. To the best of our knowledge, only the datasets in (refs 10–12) provide aggregated sampling rates above 10kSps. In contrast to the aggregate measurements, the ground truth is only available with low sampling rates, making it difficult to correlate data of individual appliances to the mains EEC with high timing accuracy (see Table 1).

Office buildings have a large potential for EEC reduction since most office workers are unaware of the energy costs they cause^26^. Modern office environments contain a well-defined set of appliances equipped with switched-mode power supplies (SMPS). Information and Communication Technology (ICT) devices, including computers, monitors, networking equipment, and battery chargers, mostly use direct current (DC) and require a power supply module. Recently, field research and trials have been conducted with buildings offering DC power sockets, removing the need for SMPSs^27,28^. Recent studies found that SMPSs can have a significant effect on EEM accuracy and can cause deviations of up to 582% when comparing smart meters to conventional meters^29^. This is primarily caused by magnetic interference due to non-linear and fast-switching loads causing distortions in current sensor readings. A significant portion of the reported errors are caused by ripple currents in the frequency range of up to 150kHz, which is currently not covered by any dataset. The authors found a significant correlation between sensor type and their measurement accuracy. While Rogowski coil-based sensors showed a positive deviation (higher readings), Hall effect-based sensors were found to predominately return negative deviations, compared to conventional electromechanical meters.

In order to study typical office appliances, in particular, ICT devices equipped with SMPSs, in the context of NIALM and EEM, we present BLOND: a Building-Level Office eNvironment Dataset. We provide long-term continuous measurements of voltage and current waveforms in a 3-phase power grid of a typical office environment collected in Germany between October 2016 and May 2017. The dataset contains readings for aggregated circuits (smart meter) and the matching fully-labeled ground truth waveform of voltage and current with a high sampling rate for individual appliances. In total, 53 appliance types and 74 appliance instances, grouped into 16 classes, are distributed across 111 recorded channels. All signal traces are precisely timestamped with a globally synchronized clock. The dataset consists of two measurement series in the same environment with different sampling rates. BLOND-50 contains 213 days of continuous readings of all 3 phases (aggregated) at 50kSps, with ground truth data (individual appliances) at 6.4kSps. BLOND-250 contains 50 days at 250kSps (aggregate) and 50kSps (individual appliances). The setup incorporates one data acquisition system for the aggregated circuits and 15 units to record individual appliances, each capable of measuring up to 6 appliances. We also provide a precomputed 1-second data summary to enable research on data with lower sampling rates.

## Methods

In order to create a new dataset focused on ICT devices equipped with SMPSs, which provides a benefit over existing public datasets, and applicable to NIALM-related areas, we define the following requirements and desirable attributes:

**High sampling rates** are necessary to extract high-frequency features from SMPS and other non-linear loads. Existing datasets (Table 1) cover the range between 10 to 20 kilosamples per second (kSps), only covering the lower region of the sampling frequency bins described in (ref. 23). New research questions can be posed with higher sampling rates, which could lead to improved accuracy and new types of algorithms.

**Ground truth waveforms** provide additional information compared to lower sampling rates (e.g., one seconds mean values). Therefore, it is beneficial to collect the per-appliance EEC with sampling rates that are capable of representing the actual mains waveform for voltage and current.

**Raw data streams** are useful if the desired information cannot easily be extracted during data collection, either because the use case is not known yet or different algorithms and filters might omit import data. This allows us to calibrate and optimize the signal quality for a given task.

**Long-term continuous recording** results in a gap-less data capture of the entire electrical circuit. Previously recorded datasets contain large gaps where simply no data was recorded or received due to various reasons. While technical systems always have a certain margin of error, integrity and completeness should be a high priority when it comes to high-frequency energy datasets.

**Clock synchronization** allows for a precise matching between aggregate and ground truth samples. The time-stamping accuracy is a side-effect of high sampling rates. Since most dataset collections happen with a distributed fleet of sensors, maintaining a precise world clock is crucial to the overall timing accuracy. Without proper synchronization, some sensors might drift in time and blur the aggregate-to-ground-truth relation.

### Environment

The BLOND dataset was collected at a typical office building in Germany, with the main occupants being academic institutes and their researchers. The measured circuits are part of a single floor with 9 dedicated offices and 160 m^2^ of office space with central (non-electric) heating. The average weekday power density over the entire measurement period was 11.7 W/m^2^-which fits into the category of typical office buildings of 9.5 to 13.5 W/m^2^ (ref. 30). Throughout the collection of the dataset the population working in the monitored offices varied from 15 to 20 people.

Periods of occupancy are closely aligned with the office work schedule in Germany: Monday to Friday with a majority of occupants being present between 9:00 and 18:00. Weekends show almost no usage of the office spaces and therefore also no electricity consumption. Major public holidays, such as Christmas and New Year, also show minimal presence in the building, as well as personal vacation days taken by occupants individually. This includes business trips, sick days, and other ‘out-of-office’ days. Due to privacy restrictions, no such data were collected.

All occupants perform light-duty office work, utilizing personal computers, monitors, and other electrical appliances typical for this environment. Individuals working in this building spend the majority of their work time at the desk, with certain breaks for meetings or other activities outside their assigned offices. Some occupants are involved in academic work and teaching, giving weekly lectures or attending meetings.

**Figure 1:**
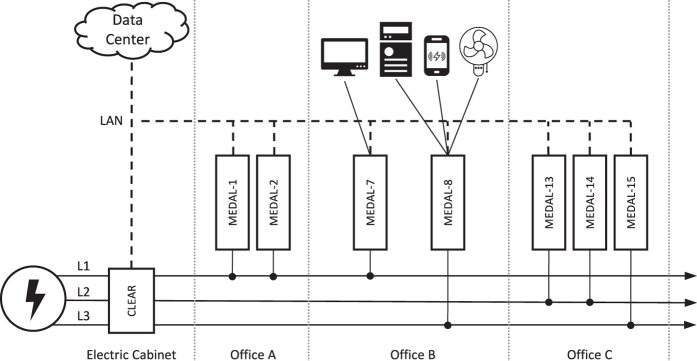
The measurement architecture of BLOND with physical placement of DAQ systems and connected appliances. A CLEAR unit is used as an EEC meter at the mains input to measure all 3 circuits in the electric cabinet. Multiple MEDAL units are placed in office rooms and connected to different circuits. Each MEDAL can be used to measure up to six appliances simultaneously in a single phase. Only a subset of MEDAL units is depicted; see Table 5 for a full circuit mapping.

The power system consists of a 50Hz mains with 3 circuits with a nominal phase shift of 120° (typical 3-phase supply): *L1*, *L2*, and *L3*. Each office room is connected to one or two circuits, with neighboring offices being on alternating circuits (see Figure 1). The building is not equipped with electric space heaters or air conditioning. Therefore, the dataset only contains user-operated appliances and base loads.

In order to keep rewiring efforts to a minimum, the existing independent circuits for regular and emergency lighting was excluded from the measurements, and only the user-accessible wall sockets were part of the measurements. The offices are electrically grouped into two sectors, each with an independent 3-phase breaker switch, resulting in 6 circuits. Since the goal of this dataset is to collect aggregated mains EEC, every two circuits per phase are combined for measurement purposes, allowing us to use a 3-phase energy data acquisition system.

### Aggregated mains measurements

Mains EEM was performed in the distribution board with a CLEAR unit^31^, which was designed to meet the BLOND requirements. CLEAR, a circuit-level energy appliance radar, is a specialized data acquisition system capable of measuring voltage and current waveforms with high sampling rates and bit-rate for a 3-phase power grid. The power necessary to operate the sensors and the CLEAR system itself is drawn from a different circuit and not part of the measurement setup.

**Figure 2:**
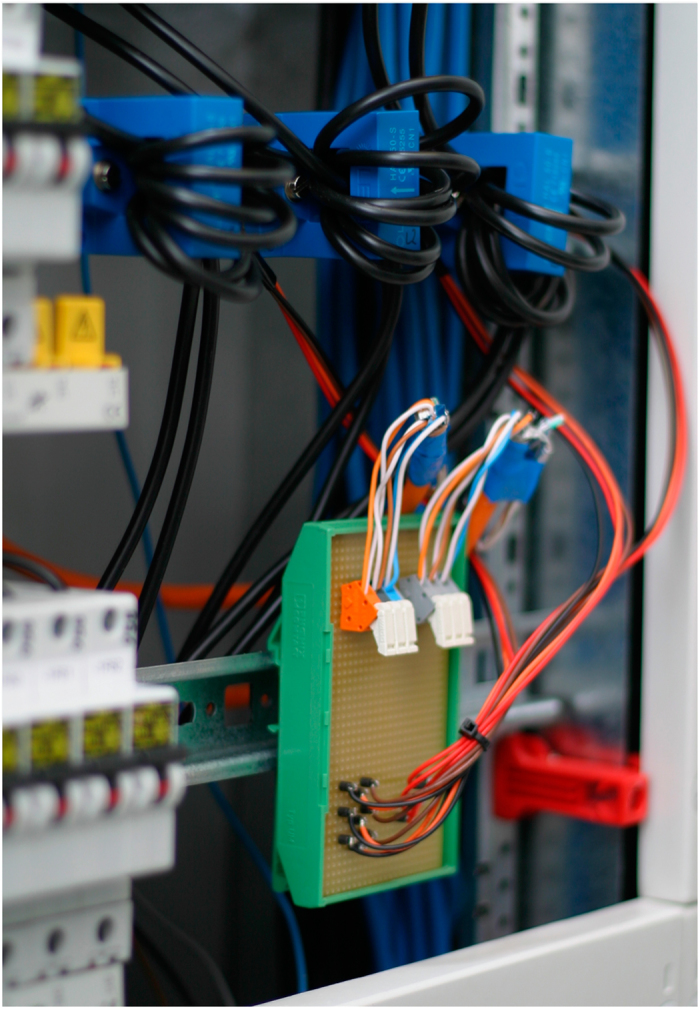
CLEAR current sensors installed in the electric cabinet. The open-loop Hall-effect sensors employ multiple turns of the mains wiring to increase the usable output signal. A small connection board distributes supply voltage and output signals. All changes and alterations were authorized and conducted by certified personnel.

The CLEAR system utilizes three Hall-effect based current sensors, installed in the electric cabinet (Figure 2), and a measurement box in the adjacent room that contains all electronics and processing units. The electric cabinet and sensors are connected to the measurement box via 2 CAT-6 cables to provide shielded signal transmission and power. The voltage signals are directly tapped off the incoming mains line.

The employed analog-to-digital converter *AD7656A* samples all six channels (3 phases: voltage and current) simultaneously with up to 250kSps (ref. 32). Each signal channel is converted with 16-bit precision and bipolar value range, allowing for a direct mapping of the AC mains waveform into a digital data stream.

The ADC is controlled by a *Lattice XO2 7000-HC* FPGA to trigger the single-shot and read the data into memory for buffering. The resulting data packets are forwarded to a USB interface chip to allow for direct communication with a single-board PC. The Linux-based single-board PC receives the data and stores it into files, which then can be sent over the network into the data center for storage.

Each circuit in each room is protected by a 16 A breaker; each mains phase is protected by a 25 A breaker. A preliminary check showed typically less than 16 A per phase of total EEC over the course of a single day. Using *LEM HAL50-S* current sensors, we can utilize 3 primary turns to boost the effective signal bandwidth without exceeding the primary nominal current of 50 A per sensor^33^. The sensors come pre-calibrated and the calibration factor (linear mapping) was computed according to the data sheet.

The voltage signal is generated by an AC-AC transformer, which depends only on the open-circuit voltage and the minimal load during measurements. The calibration factor for the voltage ADC signal was computed by taking multiple RMS readings of a calibrated high-precision voltmeter and mapping it into the ADC signal.

### Individual appliance measurements

**Figure 3:**
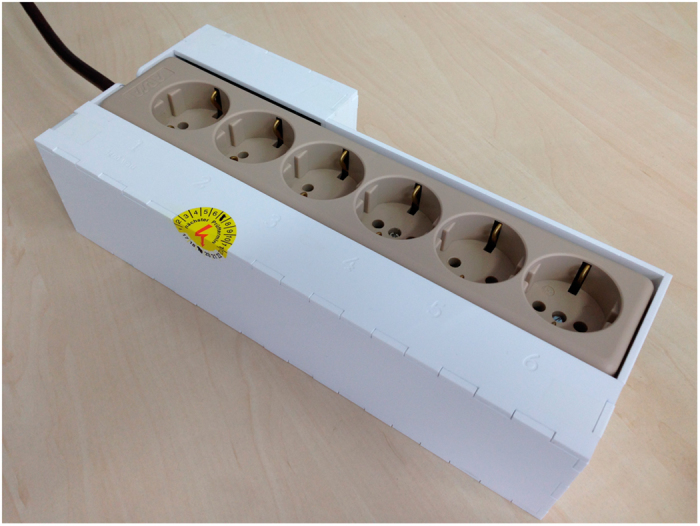
The MEDAL system used to collect ground truth appliance energy consumption data. The laser-cut acrylic enclosure contains a power strip and two boards to measure the voltage and current of each connected appliance.

The individual appliance EEM was performed by a fleet of 15 MEDAL units^34^ acting as ground truth data for the aggregated mains measurements. MEDAL, a mobile energy data acquisition laboratory, is an off-the-shelf 6-port power strip (Figure 3), augmented with voltage and current sensing infrastructure in a compact and portable enclosure. A single-board PC is used to collect EEC data from the sensing hardware and to run the same software packages as CLEAR. Therefore, the fleet of MEDAL systems and CLEAR behave identically during setup and operation.

Each MEDAL unit measures up to 6 user appliances simultaneously with labeled sockets: #1 to #6. All power sockets in the offices are directly connected to a MEDAL system, used for base load equipment, or rendered unusable to prevent unmonitored appliances from being used. All monitored energy consumption is included in the CLEAR measurements and exactly one MEDAL data stream. MEDAL uses the same voltage sensing circuit and calibration as CLEAR.

All sockets produce an independent current signal with a Hall-effect-based IC from the *Allegro ACS712* family, providing a range of 5 / 20 / 30 A_peak_ per socket. Due to the expected ICT devices with SMPSs, we chose to configure each MEDAL unit with one high-power socket (up to 3600W on socket #1), and 5 low-power sockets (up to 815W, sockets #2 through #6). The maximum safe wattage is properly marked on the enclosure next to the socket. In case the plugged-in appliance exceeds that limit, the signal is limited to the maximum value, while still being electrically safe to operate. The EEC of a MEDAL system is less than 5W and not measured in the ground truth data.

Most commonly available ADCs that offer simultaneous sampling of all channels can be expensive and are not suitable for a large-scale DAQ system. Therefore, MEDAL uses seven independent single-channel ADCs: *MCP3201* with 12-bit resolution and up to 50kSps (ref. 35). Precise timing and simultaneous sampling are achieved by using an *ATmega324PA* microcontroller as command & control IC.

### Appliance logs

An office environment with a moving and size-varying population can be an ever-changing setting to collect energy data. A list of observed appliances and their grouping into classes is available in Table 2. Most of these devices are small and portable, which means they can be moved around, plugged into different sockets, or simply appear and disappear on a daily basis. To prevent the incorrect labeling of appliance ground truth, a mapping between MEDAL sockets and actually plugged in devices was recorded in the appliance log: a spreadsheet containing timestamps, class name, appliance name, nominal power consumption, and socket numbers. The full log for each MEDAL is available in a JSON-based file format and as a spreadsheet file for easy printing and visual inspection. Although the appliance log is mostly based on self-reporting and periodic checks by trained professionals, a certain margin of error cannot be avoided. The curation of this data was carried out to the best of our capabilities and with due skill, care, and diligence.

Monthly checks were conducted to update the appliance log. Occupants were instructed to give notice about changes, so an update can be entered into the appliance log. An in-depth evaluation of the daily EEC was conducted retroactively to further improve the data quality. In cases a mismatch with the actual metered data was found, the appliance log was augmented with additional entries. This was only applicable in cases where a mismatch was deterministically resolvable by either using data from adjacent days, or by questioning the occupant responsible for the MEDAL system. Sockets marked as empty in the appliance log were manually verified by inspecting the daily EEC of the MEDAL system in question. If a mismatch was detected, the log was updated accordingly. Entries in the log dedicate one socket to one specific appliance. This does not include information about being turned on or being plugged in, but only serves as a booking.

### Data collection

BLOND aims for long-term continuous measurements, which requires some fault tolerance in the transmission layer; rendering wireless or mesh-based networks unfit for this task. The building is equipped with spare Ethernet connections in each room, which were used as a reliable transmission network to forward all data into a centralized storage system. Ethernet, IPv4, TCP, and SSH all provide mechanisms to ensure data integrity and to automatically detect and retransmit faulty data with a very high probability.

BLOND-50 employed a pull-strategy, in which a single central server periodically pulled new data files from each measurement unit and moved them into a distributed storage system. CLEAR and MEDAL convert the raw data into HDF5 files and can buffer data for multiple hours or days if nobody collects new data. The central server only has to move data between systems and also buffers data for up to 24 h in case the storage system is unavailable. This architecture decouples the various stages to allow for outages and planned maintenance. Buffer sizes and temporary storage devices were chosen carefully to maximize the allowed time before data loss occurs.

BLOND-250 uses a significant higher sampling rate, which renders a pull-strategy unusable due to memory and compute performance limits. Therefore, a push-strategy was used in which each measurement system directly sends raw data files (chunked) to the data center. The files are then converted and moved to the storage system by the server. Due to the higher sampling rate and file sizes, the available buffer time in each stage is also reduced.

CLEAR and MEDAL are built with the same software stack, which enables us to reuse large portions of the collection software and buffering strategies. Each measurement system is capable of buffering multiple gigabytes of raw data to a local storage device (SD-card or USB flash storage) in case of network failures or data center errors. This allows us to survive multiple days of data collection without any transmission capabilities. Upon reestablishing network connectivity, all buffered files are transmitted in bulk at a limited rate to prevent network congestion. Additional actions to further increase fault tolerance were implemented by using ‘RAM-first’ buffering to keep I/O access to a minimum and reduce the risk of memory wear (write endurance of NOR/NAND flash memory). Although the underlying hardware of CLEAR and MEDAL are general-purpose computing devices, some low-level measurement tasks require real-time capabilities, which have been implemented by carefully choosing data structures, in-memory buffer sizes, and I/O access patterns to guarantee error-free data collection.

All networked devices are connected to the same Ethernet and share a synchronized clock via NTP. Two stratum-3 time servers are available on the same layer-2 Ethernet. The internal system clock is connected to a dedicated real-time clock chip with a backup battery. A daemon process runs in the background to synchronize the system clock continuously; CLEAR uses *systemd-timesyncd* and MEDAL uses *ntpd*.

### Known issues

- Only user-operated appliances are measured as ground truth. Some static appliances (e.g., network switches and wireless access points) that are not user-operated are directly connected to the wall socket and can be considered as base load or static background in the CLEAR measurements (including MEDAL's own energy draw).
- MEDAL uses a unipolar ADC that can cause a slight DC-bias in the signal due to changes in the DC reference voltage. This can easily be accommodated for via proper signal calibration and filtering as part of a preprocessing stage.
- The appliance log was regularly updated and room-to-room checks were conducted. However, there could still be gaps in the log for unknown activity by students bringing their own devices for a short time period without entering the correct details into the appliance log.
- All measurement units were calibrated at the start of the BLOND data collection. Slight deviations in resistor value precision could cause a difference between CLEAR and MEDAL units connected to the same circuit.

### Code availability

We have implemented most of the data collection, technical validation, data processing, and utility tools in Python 3. The individual source files are available under the MIT license in the BLOND repository^36^.

Due to the extensive amounts of data, processing is most reasonably done in a distributed and parallelized approach. We provide usage examples that can be scaled up and run in a distributed compute environment.

Software to convert and collect measurement data from a fleet of DAQ units is provided as it was used during the BLOND data collection. All steps in the Technical Validation section can be reproduced with the supplied scripts. The 1-second data summary was created from the raw measurements, and can be fully recreated.

## Data Records

We provide raw voltage and current measurements of multiple circuits and appliances with high sampling rates. Additionally, we derived a data summary by computing various energy-related metrics into 1-second values.

### BLOND datasets

BLOND (Data Citation 1) contains two measurement series with different sampling rates:

- BLOND-50 with 50kSps (aggregate) and 6.4kSps (ground truth) over 213 days from September 30, 2016 to April 30, 2017
- BLOND-250 with 250kSps (aggregate) and 50kSps (ground truth) over 50 days from May 12, 2017 to June 30, 2017

Raw data and metadata are stored in HDF5 files that can be processed with a variety of open source and commercially available tools. Voltage and current samples of aggregate and ground truth measurements represent the waveform of the underlying electrical signal and are stored as-is from the sensor input. No permanent data cleaning or preprocessing was performed.

Metadata is embedded in each file and accessible as HDF5 attributes, either directly in the file root, or on a specific HDF5-dataset, see Table 3. Value types are either short integer, floating point, or ASCII-encoded byte strings. Generic information from HDF5 attributes matches to individual parts of the file name.

The structure of each dataset is grouped by date, and unit name into sub-directories: BLOND-50/2017-03-25/medal-6/ contains 96 files of MEDAL-6 from March 25, 2017. Files of the BLOND-250 dataset can be found in the corresponding directory. This hierarchy is also available in the associated Metadata Record (ISA-Tab). Each file name contains the unit name, date, timestamp of the first sample in the file, a timezone offset, and a sequence number: medal-6-2017-03-25T17-22-09.499845T+0100-0016925.hdf5 contains data starting roughly at 17:22 on March 25, 2017, with a timezone offset of +1 h, and this is the 16925th file in this series of MEDAL-6.

All timestamps and date information are "local time", therefore, special care must be given to the timezone offset during daylight saving time transitions: on 2016-10-30 at 3:00, DST ends (backwards 1h), on 2017-03-26 at 2:00 DST, starts (forward 1h). On December 31, 2016, a leap second was observed, which shifts back all file timestamps by one second.

Since files typically don't start at exactly 0:00 (midnight), the beginning and end of a day can be found in the previous or following file based on the sequence number.

Each measurement unit automatically splits data into chunks while the data acquisition continuous uninterrupted. The size of each chunk (number of samples per file) depends on the sampling rate and type of the unit, see Table 4. In total, BLOND consists of 945,919 files, amounting to 39TB.

### Appliance log

The appliance log is available in two file formats: appliance_log.{json, xlsx}. Both files contain the same information and can be used interchangeably. The XLSX representation is human-readable and suitable for printing, whereas the JSON data is intended as input for data processing tools.

The JSON file was created from the XLSX data with the appliance_log_json_converter.py script. It contains a list of entries for each MEDAL unit. An entry consists of a timestamp and socket declarations (one for each socket): class_name, appliance_name, and power. Every appliance instance from the appliance log is summarized in Table 2.

### 1-second data summary

Each dataset was augmented with a precomputed 1-second data summary: root-mean-square of voltage and current, real power, apparent power, power factor, and mains frequency. The resulting data was stored in one HDF5 file per day per measurement unit, covering all raw data files in each day folder (see one_second_data_summary.py). This provides quick and easy access to gain an overview of certain daily characteristics, without the need to download and process thousands of files. The daily data files are accompanied by a corresponding PDF showing selected plots of time series data, e.g., summary-2017-03-25-medal-6.hdf5 and summary-2017-03-25-medal-6.pdf.

## Technical Validation

All raw measurements included in the BLOND datasets are provided as-is, without any post-processing, cleaning, or filtering. This means the raw data must be calibrated and prepared before using the values as input to an evaluation (see the Usage Notes section). During the collection of BLOND, real world effects and noise are captured in the data. The measurement setup (environment) allowed us to have a data coverage of over 99.997% across 16 individual DAQ units during a combined period of 263 days. The missing data amounts to 2.5 h of uncovered EEC.

**Figure 4:**
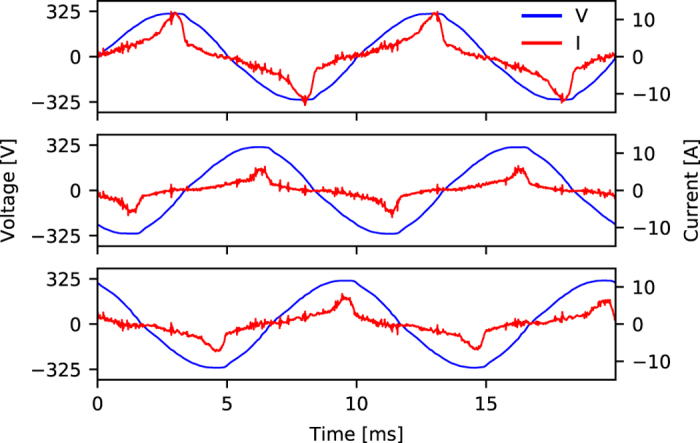
Waveforms of CLEAR circuits for voltage and current. The 3-phase power grid is characterized by a 120° phase shift between the circuits. The current consumption shows strong SMPS usage with sharp increases at each cycle apex. The voltage shows a typical sinusoidal waveform.

**Figure 5:**
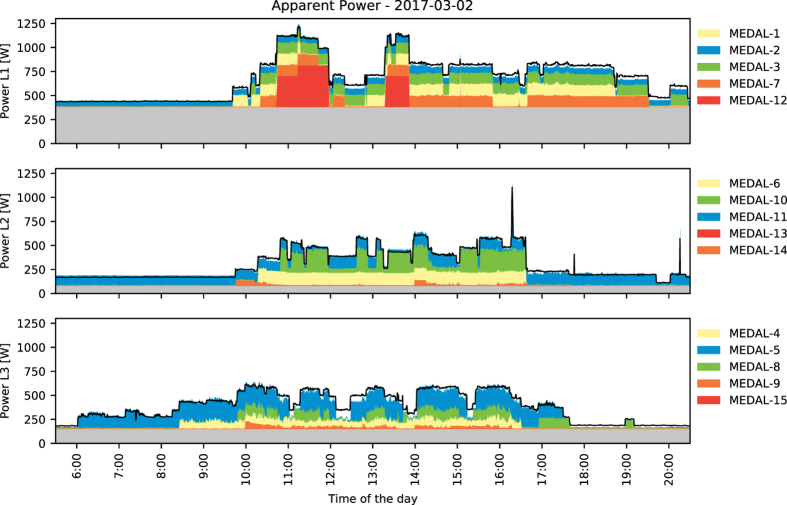
The load profile over multiple hours of the BLOND environment on 2017-03-02. Each stack plot shows the apparent power consumption of CLEAR (black line) with overlays for the contained MEDAL sub-meters (colored areas). See Table 5 for the circuit mapping. The individual steps (appliance events) match the overall load profile of the aggregate EEC. Each circuit shows a base load (gray area) which accounts for static background consumers. The 1-second data summary was downsampled to 30 s before plotting. Total consumption stays constant in the hours not shown. For visualization purposes, only the sum of all MEDAL sockets was plotted, however, the data contains an appliance-specific breakdown.

An example of the captured waveform of voltage and current signals in a 3-phase power grid with CLEAR can be seen in Figure 4. A typical load profile (with individual contributions of each measurement system) over the course of multiple hours can be seen in Figure 5. The static base load was extracted from the day-to-day offset of the total consumption for each circuit. On small time scales (multiple hours), the base load can be assumed constant; day-to-day changes can be accounted for by calibrating the static offset during the night or on weekends (constant load with no occupants). The sum of all MEDAL units matches the measured EEC with CLEAR with reasonable accuracy, however, even small voltage drops or line noise can induce errors.

### Data collection sanity checks

While collecting data, each DAQ unit performs sanity checks for each new data chunk. This includes a DAQ continuity and generic transmission error checks. Such errors could be caused by internal queues filling up, full USB transfer queues, or interrupted communication between components. Each chunk contains a sequential identifier that can be validated to match its immediate predecessor and successor. In the case a mismatch is detected, the acquisition stops, reinitializes all components and retries. These identifiers are available in every HDF5 file for offline verification (*trigger ids*). No errors were detected during the collection of the BLOND datasets.

Complementing this low-level check, each newly created HDF5 file gets assigned an increasing sequence number, which marks a continuous uninterrupted series. BLOND-50 and BLOND-250 consist of a single long-term measurement series for each DAQ unit. Only one interruption was detected: the CLEAR unit in BLOND-50 on 2016-10-18 (last sequence number: 0005172), due to a manual reboot after installing security updates. The gap covers only CLEAR measurements for 2 h, 19 min, and 27 s. MEDAL measurements were not affected.

### Sampling rate precision

Each data acquisition system collects data with a fixed sampling rate. An internal oscillator serves as a precise clock generator to trigger each analog-to-digital conversion. Depending on environmental factors, this process experiences a small unpredictable shift in speed. The actual average sampling rate was calculated based on the timestamps (with NTP precision) of the first and last data file over a 24 h period (see average_sampling_rate.py) since all files contain the same amount of data (samples).

**Figure 6:**
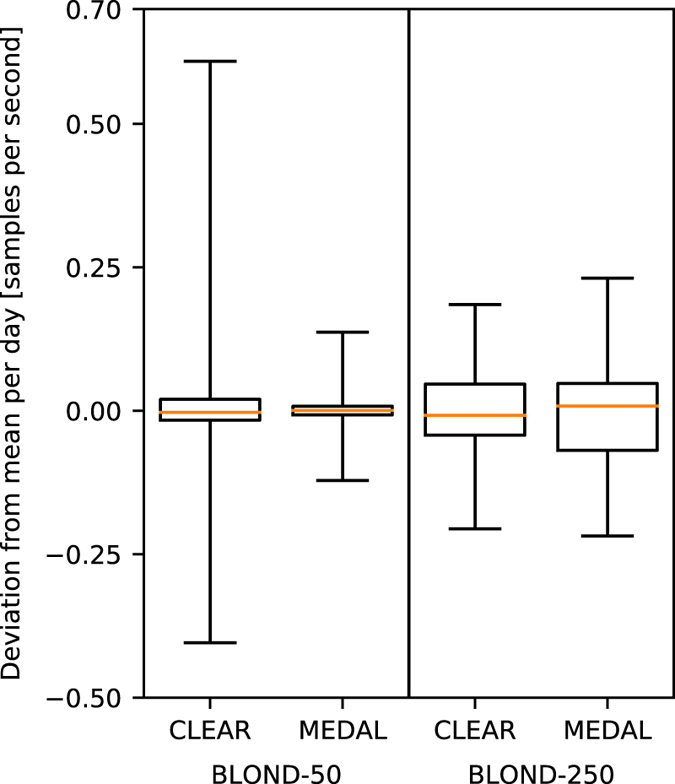
Boxplot of sampling rate precision per day. This boxplot shows the distribution of average sampling rates and its variation over 213 / 50 days for each measurement system. The whiskers depict the minimum and maximum. The mean was subtracted from each entry to compare only the daily fluctuations. The median is shown as line in the box.

The average sampling rate per day shows an almost constant offset of less than 0.5%, while the actual variations are smaller than 1Sps over the course of 24 h, see Figure 6.

For BLOND-50, CLEAR has a nominal sampling rate of 50000Sps, mean of 49952.355, and a standard deviation of 0.057. All MEDAL units combined have a nominal sampling rate of 6400Sps, mean of 6399.880, and a standard deviation of 0.013.

For BLOND-250, CLEAR has a nominal sampling rate of 250000Sps, mean of 248767.169, and a standard deviation of 0.084. All MEDAL units have a nominal sampling rate of 50000Sps, mean of 49984.059, and a standard deviation of 0.092.

### Clock synchronization

All timestamps used for marking samples and the beginning of new file chunks are derived from the system clock of the single-board PC in each measurement unit (CLEAR and MEDAL). The NTP precision as reported by ntpq -c rl is −20, yielding a theoretical timing accuracy of 0.95µs. The real-world delay, offset, and jitter values of ntpq -p show an average of 1ms to 2ms.

The sampled values from the ADC are buffered and transmitted via USB bulk transfers. The timestamp is added on the host device (single-board PC), which could add a short delay between the actual sampling time and the time it gets timestamped. Using USB data packets of 510 bytes containing 36 samples, the resulting average time jitter is 2.81ms at 6.4kSps, and 0.72ms at 250kSps. The preemption latency for CPU-bound tasks is defined with 6ms (Linux kernel v4.4.21).

To verify these theoretical values, we used a space heater to generate a visible appliance switch-on event in socket 1 of MEDAL-1 on 2017-06-12 at timestamp 11:10:58. The difference of the sharp transient in the CLEAR to the MEDAL time series data was measured with 6.8ms, which is within our estimation (see clock_synchronization.py). This allows us to synchronize multiple data streams with sub-cycle precision on a 50Hz mains.

### Per-file data checks

The correctness of the sampled voltage and current signals was validated by analyzing each data file with 15 individual checks to assert various metrics and raw data streams (see per_file_data_checks.py):

**Dataset length** is the amount of samples per signal in a given file. This is defined by the sampling rate and the file size used for chunking. If the data acquisition is briefly interrupted, stopped, or the file got truncated, the expected length does not match. In BLOND-50, 4 individual files were found that failed this check. The data collection seems to have continued, however, these files were either corrupted during transmission or the storage system failed to persist the data. The files in question have a length of 0 bytes and are not valid HDF5 files. MEDAL 6, 13, and 14 at sequence number 0016123, as well as CLEAR at 0043125 are affected.

**Mains frequency** is expected to be 50Hz and should only deviate slightly. The mains frequency was computed using Fast Fourier Transform and selecting the strongest bin. Erroneous readings would indicate a collapsing power grid or a malfunctioning ADC trigger input (sampling rate). No such errors were found.

**Voltage and current root-mean-squared** expected values are based on the measurement unit capabilities and can be used as a sanity check to check against unexpected high or low values. Voltage values must be close to 230V_RMS_, have an almost zero absolute mean, and the crest factor should be around 1.41. Current values must be below the rated measurement limit of the DAQ unit, have an almost zero absolute mean, and the crest factor must be greater than 1.2. No such errors were found.

**Raw voltage value range and bandwidth** is defined by the ADC bit-resolution. A 16-bit ADC can yield up to 65536 different measurement values. If the measurement range was calibrated or configured incorrectly, not all values would be used, resulting in degraded accuracy. We checked how many unique values are present in each signal per file and compared the maximum to the minimum, which must be within certain threshold limits. No such errors were found.

**Voltage bandwidth** is defined by the power grid; for BLOND, we expect a nominal voltage of ± 324V_peak_. Including a certain margin of deviation that is allowed during normal operation of the grid, we checked the minimum and maximum voltage values to be within certain threshold limits. No such errors were found.

**Flat regions** are defined as intervals with identical consecutive values. During initial experiments and prototyping, we detected a malfunction in one of the traces on a PCB. This led to a permanently pulled-low bit on a data bus. The DAQ unit therefore only received the same value over and over again. We checked for flat regions longer than a certain threshold by applying a linear convolution with a filter kernel (length of one mains period) to each signal per file. No such errors were found.

## Usage Notes

The BLOND data files are provided in HDF5 format, which is usable in most scientific computing packages, e.g., Python (h5py/numpy/scipy), MATLAB (h5read), R (rhdf5), Mathematica (Import), and NILMTK^37^. The metadata (HDF5 attributes) is documented in Table 3. Each HDF5 dataset was created with the following filters: gzip compression (reduces file size), shuffle (improves compression ratio), and Fletcher (adds checksums to detect data corruption). HDF5 offers a multitude of different filters with potentially better compression, however, we wanted to retain compatibility with most software packages, which typically lack support for 3rd-party filters.

Multiple example use cases for data handling and calibration can be found in the provided source code. We recommend performing a mean-offset normalization for each mains cycle before multiplying the signal with the calibration factor to remove any unwanted DC-biases in MEDAL signals. We deliberately did not clean, back-fill, or strip any of the data. This allows us to retain and extract as much information as possible from seemingly ‘empty’ data (background noise, sampling artifacts, derived data).

## Additional information

**How to cite this article**: Kriechbaumer, T. & Jacobsen, H.-A. BLOND, a building-level office environment dataset of typical electrical appliances. *Sci. Data* 5:180048 doi: 10.1038/sdata.2018.48 (2018).

**Publisher’s note**: Springer Nature remains neutral with regard to jurisdictional claims in published maps and institutional affiliations.

## Supplementary Material



## Figures and Tables

**Table 1:** Overview of long-term energy datasets with high sampling rates.

**Dataset**	**Circuits Phases**	**Aggregate Sampling**	**Ground Truth Sampling**	**Duration**	**Classes**	**Appliances**
REDD^10^BLUED^11^UK-DALE^12^	221	15000 Sps12000 Sps16000 Sps	1 Sps1 Sps$0.1\dot{6}$ Sps	119 days7 days655 days	8916	824353
BLOND-50BLOND-250	33	50000 Sps250000 Sps	6400 Sps50000 Sps	213 days50 days	1616	5353
This includes only datasets with long-term recordings of aggregate (above 10kSps) and per-appliance measurements. In contrast to existing datasets, BLOND also provides ground truth data with a high sampling rate.						

**Table 2:** List of appliances observed in the BLOND dataset.

**Class**	**Manufacturer**	**Type**	**Power**	**Count**
Battery Charger	Kraftmax	BC4000 Pro	18 W	1
	DJI	Phantom 3	100 W	1
Daylight	Philips	HF3430	10 W	1
Desktop Computer	*generic*	Intel Xeon E5-1640 v4, NVIDIA TITAN X	1200 W	1
	Dell	OptiPlex 7040	65 W	1
	Dell	OptiPlex 9020	65 W	2
	Dell	T3600	635 W	1
Dev Board	FPGA	Xilinx ML505	30 W	1
	FPGA	Tegra Jetson	90 W	1
	MEDAL	Prototype	5 W	1
Electric Toothbrush	*generic*	inductive charging	5 W	1
Fan	Eurom	VS 16	45 W	1
	VOV	VTS-1641	50 W	1
Kettle	Clatronic	WK3445	2000 W	1
	Severin	WK3364	1800 W	1
Laptop Computer	Apple	MacBook Air 13'' Early-2014	45 W	1
	Apple	MacBook Pro 13'' Mid-2014	60 W	3
	Apple	MacBook Pro 15'' Mid-2014	85 W	2
	ASUS	N750JV	120 W	1
	Dell	E6540	130 W	1
	Dell	XPS13	45 W	1
	Lenovo	Carbon X1	90 W	1
	Lenovo	B560	65 W	1
	Lenovo	L540	90 W	1
	Lenovo	T420	90 W	1
	Lenovo	T450	65 W	1
	Lenovo	T530	90 W	1
	Lenovo	X230 i7	65 W	1
	Lenovo	X230 i5	170 W	1
	Schenker	W502	180 W	1
	Sony	Vaio VGN FW54M	92 W	1
	*generic*	SMPS, 19V	100 W	1
Monitor	Dell	P2210	22 W	1
	Dell	U2711	133 W	6
	Dell	U2713Hb	130 W	8
	Dell	UP2716D	45 W	2
	Fujitsu-Siemens	P17-1	36 W	1
Multi-Tool	Mannesmann	M92577	135 W	1
Paper Shredder	HSM	Shredstar	250 W	1
Printer	HP	LaserJet Pro 400	425 W	1
Projector	Epson	EB-65950	450 W	1
Screen Motor	Projecta	DC 485	210 W	1
Space Heater	Heller	ASY 1507	1500 W	1
USB Charger	*generic*	single USB power adapter	10 W	2
	inateck	UC2001	15 W	1
	Aukru	BS-522	20 W	1
	Apple	MD836ZM EU	12 W	1
	Apple	MD813ZM EU	5 W	2
	Chromecast	single USB power adapter	10 W	1
	Hama	00091321	10 W	1
	Samsung	Travel	10 W	3
	Sony	single USB power adapter	10 W	1
	Sony Ericsson	EP 800	10 W	1
This list was extracted from the appliance log and contains all devices used in the BLOND environment. A class label was assigned to group similar appliances. The manufacturer, type, and power information was taken from an attached name plate (if available) or the suppliers datasheet.				

**Table 3:** HDF5 dataset file metadata.

**Path**	**Attribute**	**Description**
/	name	Name of the measurement unit
/	year	Year of the first sample in this file
/	month	Month of the first sample in this file
/	day	Day of the first sample in this file
/	hours	Hours of the first sample in this file
/	minutes	Minutes of the first sample in this file
/	seconds	Seconds of the first sample in this file
/	microseconds	Microseconds of the first sample in this file
/	sequence	Sequence number in this measurement series
/	timezone	Timezone offset (daylight saving time)
/	frequency	Nominal sampling rate in
/	first_trigger_id	Internal trigger number to detect gaps
/	last_trigger_id	Internal trigger number to detect gaps
/<dataset>	calibration_factor	Multiplication factor for calibration to get or
/<dataset>	removed_offset	Removed DC-offset of the signal
/	average_frequency	Average sampling rate over 24 h
/	delay_after_midnight	Delay in seconds after 00:00
Each attribute is accessible via a HDF5-attribute-path.		
Values are provided in base units (Volt, Ampere, Hertz). Some attributes are only available in the 1-second data summary.		

**Table 4:** File chunking and length.

**Dataset**	**Unit Type**	**Sampling Rate**	**File Length**	**Samples**
BLOND-50	CLEARMEDAL	50 kSps6.4 kSps	5 min15 min	15,000,0005,760,000
BLOND-250	CLEARMEDAL	250 kSps50 kSps	2 min2 min	30,000,0006,000,000
BLOND-50 and BLOND-250 use different file sizes to chunk the continuous data stream. The size depends on the available computing resources in each DAQ unit and the configured sampling rate. The final size of the HDF5 only depends on the number of samples and the achievable compression ratio.				

**Table 5:** Circuit mapping for each measurement system.

**CLEAR**	**MEDAL**
L1	1, 2, 3, 7, 12
L2	6, 10, 11, 13, 14
L3	4, 5, 8, 9, 15
Associating a MEDAL unit with its aggregated circuit in the CLEAR data is fixed and does not change over time. This corresponds to the wiring of individual office rooms to use one (or more) of three different phases.	
